# Authentication of Counterfeit Electronics Using Rapid THz Time-of-Flight Imaging

**DOI:** 10.3390/s25165160

**Published:** 2025-08-19

**Authors:** Hyeonseung Ryu, Byung Hee Son, Jihwan Kim, Jangsun Kim, Yeong Hwan Ahn

**Affiliations:** 1 Department of Physics and Department of Energy Systems Research, Ajou University, Suwon 16499, Republic of Korea; rhw5427@ajou.ac.kr (H.R.); bhlove85@ajou.ac.kr (B.H.S.); kgw1021@ajou.ac.kr (J.K.); 2 Panoptics Corp., Seongnam 13516, Republic of Korea; jskim@panoptics.net

**Keywords:** terahertz imaging, authentication, nondestructive testing, time-of-flight

## Abstract

In this study, we used rapid terahertz (THz) time-of-flight (ToF) imaging to identify counterfeit electronics prevalent in the online market. THz-ToF allows the nondestructive reflection imaging of portable electronic devices enclosed by plastic cases with a spatial resolution of 1 mm and a depth range larger than 5 mm. For instance, we could identify a counterfeit solid-state device because there was a striking difference in the internal structure compared with that of an authentic device, although its appearance was similar. It is also possible to authenticate small portable devices with arbitrary shapes, such as earphones; THz-ToF imaging can be applied to authentication from a variety of perspectives. Importantly, our technique is particularly useful for identifying the inappropriate use of integrated circuit (IC) chips, such as controller chips in USB hub devices. THz-TDS images for various portable and wearable devices will be useful for rapid in-line authentication of products and will offer an effective tool for identifying fake electronics and the inappropriate use of IC chips in various equipment.

## 1. Introduction

The rapid growth of the digital age and e-commerce has increased the demand for sophisticated electronic products, such as wearable devices and data storage media. However, counterfeit electronic products, causing economic losses, brand damage, and safety concerns, have proliferated, posing serious risks to both manufacturers and consumers [1,2,3]. Therefore, effective nondestructive methods should be developed to identify counterfeit goods. Several traditional methods for counterfeit detection, such as visual inspection, radio-frequency identification (RFID) tags, and destructive testing, have significant limitations [4]. For instance, visual inspection often lacks precision, and RFID systems are susceptible to cloning or manipulation [5]. In contrast, destructive testing, which involves disassembling a device for inspection, may damage the product or expensive components may be lost. X-ray inspection provides a nondestructive alternative, allowing thorough examination of internal structures without dismantling the product [6]. Therefore, this method could help identify fake devices. However, X-ray inspection has its limitations because X-rays can damage electronic devices, leading to malfunctions or reduced performance. In addition, prolonged exposure to X-ray radiation may pose health risks to operators if adequate safety measures are not implemented [7]. Other nondestructive alternatives also exhibit inherent limitations. For instance, infrared thermography relies solely on surface thermal contrasts and cannot detect subsurface defects in plastics due to rapid heat diffusion [8]. Ultrasonic testing requires liquid or gel couplants [9]; optical coherence tomography is limited to shallow penetration depths of a couple of millimeters and cannot maintain the high scan rates needed for rapid, large-area 3D inspections [10,11].

Terahertz (THz) spectroscopy has emerged as an effective tool for nondestructive testing (NDT), including device inspection, biomedical diagnosis, and material characterization [12,13,14,15,16,17,18,19,20,21,22,23,24]. As THz waves can penetrate nonconductive materials, THz imaging is particularly useful for characterizing internal structures. Continuous-wave THz imaging offers real-time imaging capabilities essential for applications requiring immediate analysis and feedback [25,26,27,28,29,30,31,32,33,34,35,36]. Furthermore, time-domain spectroscopy (TDS) enables the collection of high-resolution depth and range information without the need for frequency sweeping [37,38,39,40,41,42,43,44,45,46,47,48,49]. Through the time-of-flight (ToF) of reflected THz pulses, THz pulsed imaging can obtain direct 3D mapping of objects, with a longitudinal resolution of up to 100 μm, depending upon the temporal width of the pulse source [50]. In this context, THz imaging has gained significant attention as a promising alternative to noninvasive, high-resolution structural analysis [51]. Recent advancements in THz technology have significantly improved the sensitivity and resolution of spectroscopic measurements, and innovations such as enhanced THz detectors and sources have enabled more precise and faster inspections. In addition, compressed sensing strategies have reduced acquisition times by reconstructing images from highly undersampled data [52,53], while deep learning–based reconstruction algorithms have further enhanced image quality and noise robustness [54,55]. However, THz imaging has not been considered for NDT applications to identify fake electronic devices. In particular, rapid imaging has not been demonstrated, which is crucial for practical application in everyday lives and, additionally, for the in-line inspection of commercialized products [56,57,58,59].

In this study, for the first time, we used rapid THz-ToF systems to identify false electronic devices available in the market. NDT images of commercial electronic storage devices, portable devices, and electronic controller chips were obtained. In a direct comparison with authentic devices, we identified fake electronic devices accurately and rapidly for potential use in the in-line inspection of products.

## 2. Experimental Setup

**Figure 1:**
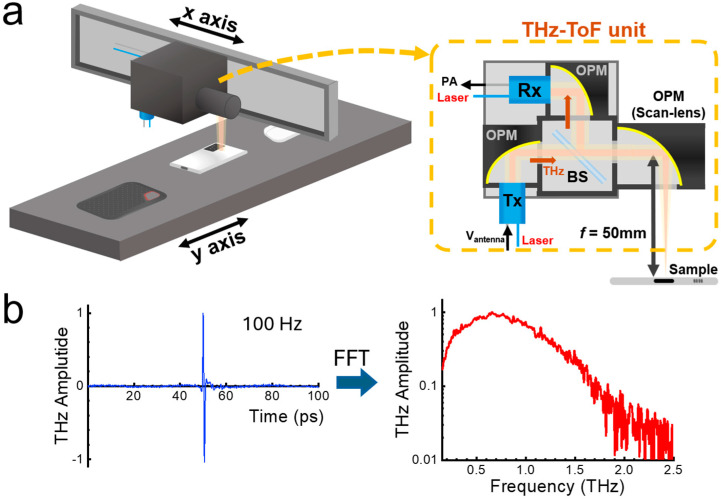
(**a**) Schematic of rapid THz-ToF imaging setup in the reflection geometry. The image was measured at 100 Hz/pixel based on ASOPS technology (OPM: off-axis parabolic mirror, Tx: THz emitter, Rx: THz receiver, BS: beamsplitter, PA: preamplifier). (**b**) Representative time- and spectrum-domain THz amplitudes measured in the reflection geometry (FFT: fast Fourier transformation).

Figure 1 shows a schematic of the THz-ToF imaging setup used for authentication. This was accomplished using a commercially available asynchronous optical sampling (ASOPS) system (TERA-ASOPS; Menlo Systems GmbH, Martinsried, Germany) which consists of two independent femtosecond fiber lasers operating at 1.56 μm, synchronized with each other. The repetition rate of one laser is fixed at *f*_0_ = 100 MHz, and the other is *f*_0_ + Δ*f*, in which Δ*f* determines the scan rate of the system [44,50,60]. This ASOPS system offers a full time delay of 10 ns at a scan rate of Δ*f* = 100–500 Hz. In our experiment, the typical time-delay range was 100 ps. Here, the time delay can be converted into depth information, where 1 ps corresponds to 0.15/*n* mm and *n* is the refractive index of the material. The scan rate was maintained at 100 Hz per pixel; conversely, we used 20–50 Hz per pixel by applying point averaging when we needed an improved signal-to-noise ratio. In addition, we note that imaging large-scale samples (e.g., >50 mm in width) has large inter-pixel distances; the scan speed was reduced to ensure stable operation of motorized stages.

A scan lens with a diameter of 25 mm and a focal length of 50 mm (Thorlabs Corp., Newton, NJ, USA) was used to focus the THz beam onto the samples. The lateral resolution of our ToF imaging was 1 mm when we used a 50 mm scan lens. The depth of focus (DOF) reached 10 mm, which is sufficient for many practical authentication applications; conversely, higher spatial resolution is possible with the short focal-length optics with the limited DOF. In the *x*-direction, the THz-ToF unit was moved forward and backward by using motorized stages (DL225; Newport Corp., Irvine, CA, USA), whereas in the *y*-direction, the sample was raster-scanned. The THz-ToF unit contained an emission antenna (Tx), a detection antenna (Rx), a 50:50 beam splitter (BS), and a scan lens. With fast scanning on the *x*-axis and slow scanning on the *y*-axis, we maintained the speed limited by the ASOPS THz-TDS system. Homebuilt analysis software was used to reconstruct images from a single binary file. The phase-sensitive THz amplitudes can be converted into envelope signals using the Hilbert transformation from the complex THz signal E˜THz=Re(E˜THz)+iH[Re(E˜THz)], where *H* is the Hilbert transformation [61]. Hilbert transformation is useful to extract envelope and phase information out of the phase-sensitive THz-TDS signals; in other words, we obtain the envelope signal, $\left|{\tilde{E}}_{\mathrm{THz}}\right|$, as a function of *x*, *y*, and *T*.

## 3. Results

**Figure 2:**
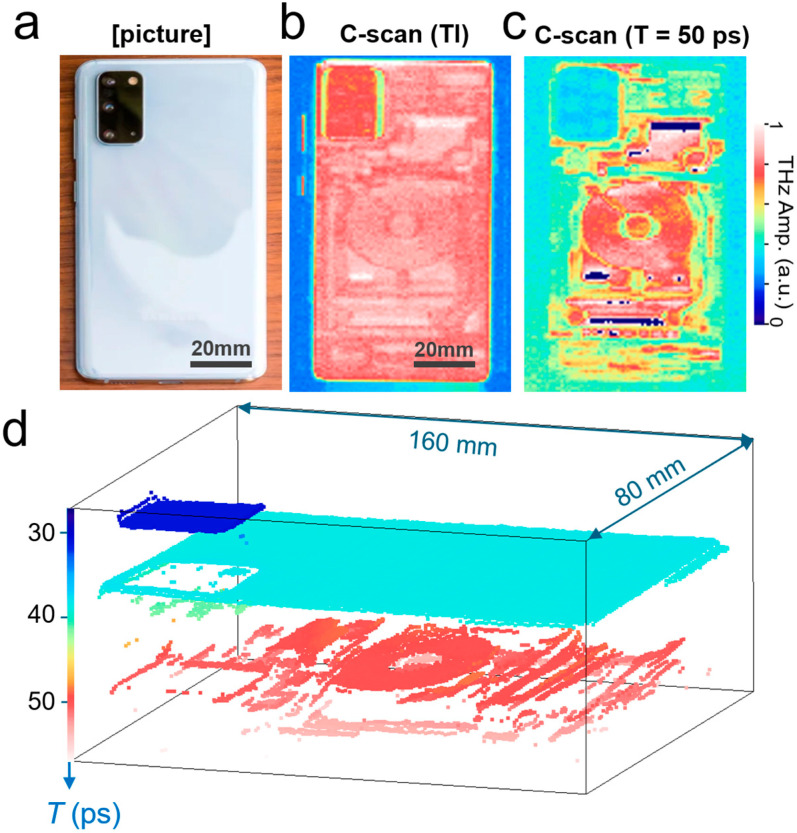
(**a**) Photograph of a smartphone viewed from the rear side. (**b**) Time-integrated C-scan image for the reflection amplitude (scale bar: 20 mm). The image size was 80 × 160 mm^2^, with a pixel size of 80 × 160 pxl^2^. (**c**) C-scan amplitude image at *T* = 50 ps. (**d**) Reconstructed 3D image from THz-ToF data.

To demonstrate the usefulness of our system, we illustrated representative THz nondestructive imaging on an electronic device, as shown in Figure 2a–c. A photograph of the smartphone (Samsung Galaxy S20+; Samsung Electronics Co., Suwon, Republic of Korea) used for the THz imaging is shown in Figure 2a. The image size was 80 × 160 mm^2^, with a pixel size of 80 × 160 pxl^2^. Figure 2b illustrates a time-integrated C-scan image (as a function of the *x*- and *y*-axes) for the THz reflection amplitude signals. In other words, signals from the entire depth range were added. In addition, we captured another C-scan image at *T* = 50 ps, as shown in Figure 2c. The internal structures hidden beneath the plastic enclosure were identified.

The signals were reconstructed into a 3D image, as shown in Figure 2d, where the vertical location of the reflected THz waves at each pixel is displayed. The image is reconstructed in 3D by plotting the peak position in the *T*-domain by fitting the envelope function at each pixel signal. The peak position is displayed when it is above a threshold value. Here, the color scale indicates the time-delay *T* at which reflection occurs. We plotted the images over a time span of 30 ps, which corresponds to a free-space traveling distance of 4.5 mm. The top enclosure and internal structures, including the wireless charging antenna, were visible. As will be demonstrated below, manufacturers usually concentrate on the external appearance of counterfeit electronic devices while neglecting internal structures. It is, therefore, possible to quickly identify counterfeit devices using THz-NDT imaging.

**Figure 3:**
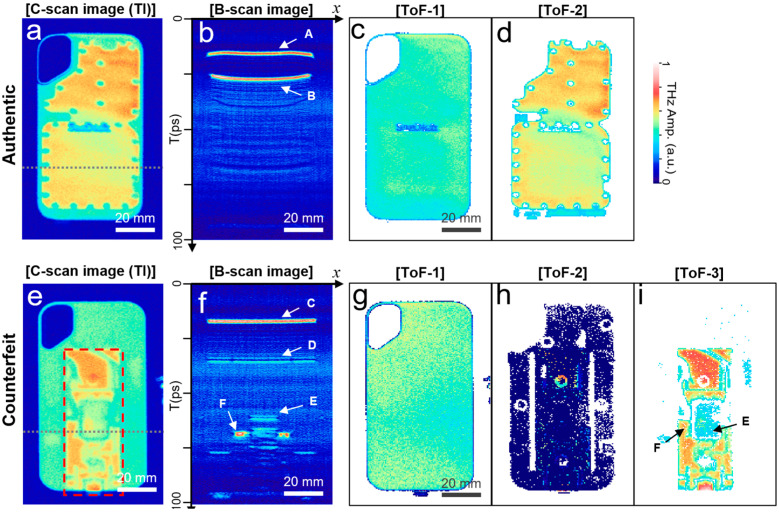
THz-ToF imaging results for an authentic SSD with time-integrated C-scan image (**a**), B-scan image along the dotted line (**b**), ToF-1 image (**c**), and ToF-2 image (**d**). The image size was 60 × 100 mm^2^, with a pixel size of 120 × 200 pxl^2^. THz-ToF results for the counterfeit SSD with time-integrated C-scan image (**e**), B-scan image along the dotted line (**f**), ToF-1 (**g**), ToF-2 (**h**), and ToF-3 (**i**) images.

A representative example of counterfeit device identification is shown in Figure 3. There is a plethora of fake storage devices in the online market. We purchased a portable solid-state device (SSD) that ostensibly offers 2 TB of memory for just 20–30 USD. As we tested the device, it delivered only approximately 50 GB of memory with an extremely low file transfer rate. We also prepared an authentic SSD with almost the same appearance for a comparison (SanDisk Extreme PRO Portable SSD; SanDisk Corp., Milpitas, CA, USA). Figure 3a shows the THz-ToF results for the authentic device using a time-integrated (TI) C-scan image. The image size was 60 × 100 mm^2^, with a pixel size of 120 × 200 pxl^2^. The rear side of the device was scanned because it had an embossing pattern on the front surface, which distorted the THz images. Figure 3b shows a B-scan image (i.e., as a function of the *x*-axis and time delay *T*) taken along the dotted line in Figure 3a. It is characterized by two strong reflection surfaces: one from the top enclosure (denoted by **A**) and the other from the backplane of the circuit boards (denoted by **B**) for SSD storage electronics. The series of images in Figure 3c,d are the ToF-1 and ToF-2 images, which represent the reflection amplitudes from the 1st and 2nd reflecting surfaces at each pixel, respectively [50]. These ToF images are an effective alternative to the C-scan images taken at the specific time delay *T* (as shown by Figure 2c). This is particularly useful for the NDT of the internal structure when information on the vertical location of interest is missing or when the surfaces are tilted. Specifically, ToF-1 represents the top surface of the enclosure, whereas ToF-2 indicates reflection from the bottom surface of the circuit.

Conversely, the ToF imaging results from the counterfeit devices (Figure 3e–i) exhibited different characteristics, as shown by a series of images with TI C-scan (e), B-scan (f), ToF-1 (g), ToF-2 (h), and ToF-3 (i). First, the counterfeit device is distinguishable from the C-scan image because it is apparently far from the authentic one. In the B-scan image shown in Figure 3f, multiple layers appear along the dotted line, in contrast to the authentic device. Specifically, there was a reflection from the front side of the enclosure (denoted by **C**), followed by that from its rear side (denoted by **D**). Furthermore, we identified the top surface of the integrated circuit (IC) chip at *T* = 65 ps (denoted by **E**) in the central region, and the top surface of the printed circuit board (PCB) was also visible (denoted by **F**). The series of ToF images (i.e., for the 1st, 2nd, and 3rd reflection surfaces, respectively, shown in Figure 3g–i) show consistent results expected from the B-scan image. In particular, in Figure 3i, there is a PCB, and the IC chip in the middle is visible, which is consistent with the C-scan image in Figure 3e, denoted by the rectangular shape with a red dashed line.

**Figure 4:**
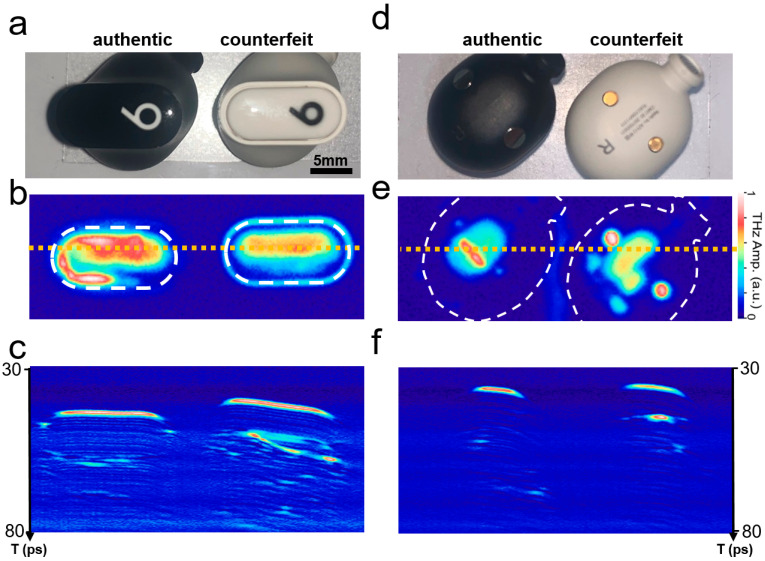
(**a**) Front-side photograph of the authentic (left) and counterfeit (right) earphones (scale bar: 5 mm) and corresponding time-integrated C-scan (**b**) and B-scan (**c**) images. The image size was 40 × 15 mm^2^, with a pixel size of 200 × 75 pxl^2^. (**d**) Rear-side photograph of the authentic (left) and counterfeit (right) earphones and corresponding time-integrated C-scan (**e**) and B-scan along dotted line (**f**) images.

Another example of counterfeit device identification is shown in Figure 4. Earphones are popular portable electronic devices that are indispensable in everyday life and are widely counterfeited by online retailers. Common signs of fake earphones include poor build quality, such as flimsy materials and loose connections, and sound quality that is often noticeably inferior, with distortion or imbalance in audio. Figure 4a shows pictures of authentic (Beats Studio Buds+; Apple Inc., Cupertino, CA, USA, left) and counterfeit (right) earphones purchased online. Their appearances are similar; therefore, careful examination is necessary to identify counterfeits. The TI C-scan image in Figure 4b illustrates the rapid identification by THz imaging. The image size was 40 × 15 mm^2^, with a pixel size of 200 × 75 pxl^2^. There is a marked difference between authentic and counterfeit devices, without needing detailed information regarding their internal structures. Conversely, the B-scan image in Figure 4c along the dotted line (where there is a large portion of reflected signals) is illustrated in Figure 4b. The cross-sectional images of the counterfeit device show a pattern different from that of the authentic one, where the pattern of the counterfeit one is slightly tilted.

As mentioned earlier, the ASOPS technique allows the inspection of a large depth range, in principle, determined by a repetition rate of 100 MHz, that is, by an interpulse duration of 10 ns [60]. This corresponds to a full-range depth range of 1.5 m when traveling through air. However, in reality, this is determined by the depth of focus, which reached approximately 7 mm under our experimental conditions when we used a scan lens of 50 mm and an input beam size of 20 mm [62,63]. In addition, the effective range is limited by the types of materials and structures of objects. As shown in the B-scan images in Figure 4c, the effective depth range is approximately 4 mm, and further details cannot be identified in this image. However, inspection can be performed from different view angles when necessary, as shown in the picture (d), C-scan (e), and B-scan (f). The differences between them can be identified from the THz-ToF images, enabling rapid earphone authentication. In other words, orientation-independent inspection is possible using the THz-ToF approach, making it an ideal tool for rapid authentication. In many cases, the authentication applications require data from authentic devices as shown in Figure 4. Therefore, a database of THz images for various portable and wearable devices would benefit rapid in-line authentication; furthermore, it would reduce the number of pixels needed for identification.

**Figure 5:**
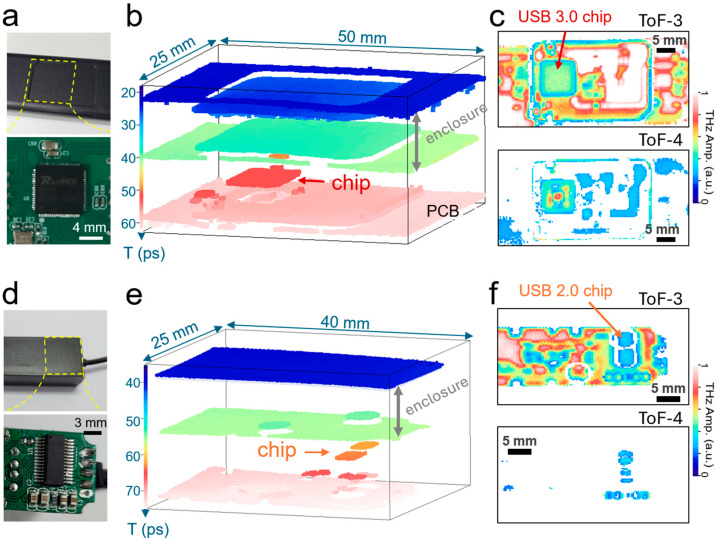
(**a**) Photograph of a USB 3.0 hub device. (bottom) Photograph showing the circuit board with a USB 3.0 controller chip. (**b**) 3D image for the device shown in (**a**), reconstructed with THz-ToF imaging. (**c**) ToF-3 (top) and ToF-4 (bottom) images with an arrow indicating the USB 3.0 chip. The image size was 50 × 25 mm^2^, with a pixel size of 150 × 75 pxl^2^. (**d**) Photograph of a USB 2.0 hub device. (bottom) Photograph showing the circuit board with a USB 2.0 controller chip. (**e**) 3D image for the device shown in (**d**), reconstructed with THz-ToF imaging. (**f**) ToF-3 (top) and ToF-4 (bottom) images with an arrow indicating the USB 2.0 chip. The image size was 40 × 25 mm^2^, with a pixel size of 120 × 75 pxl^2^.

Finally, we demonstrated the identification of the inappropriate use of the controller chip in small electronic devices, such as USB hub devices. In counterfeit electronics, cheaper controller chips are occasionally used to reduce manufacturing costs [64]. Special care is required to reduce potential damage; however, in many cases, it is difficult to discover this misconduct before disassembling items. However, THz-ToF imaging with a large depth range offers an ideal tool for identifying this phenomenon. Figure 5 shows a representative example; devices with the USB 3.0 protocol deliver speeds more than 10 times faster than USB 2.0; the controller chip is more expensive for USB 3.0. Counterfeit devices exist in which the controller chip for USB 2.0 is used for the USB hub with the appearance of USB 3.0 (painted blue in the port).

In general, the USB 3.0 controller chip employs Quad Flat No-lead (QFN) packages, such as QFN 64 (64 pin), to enhance thermal dissipation, improve electrical performance, enable miniaturization, and support a higher pin count [65,66]. By contrast, low-cost USB 2.0 controller chips predominantly use Small Outline Package (SOP) designs, such as SOP 16 (16 pin), valued for their lower manufacturing costs, legacy compatibility, and simplified circuit configurations while featuring fewer pins. These packaging differences result in distinct variations in the form factor, size, and functional capabilities of the two chips. In particular, the USB 3.0 chip has a square shape with 8 × 8 mm^2^, whereas the USB 2.0 chip has typical dimensions of 4 × 10 mm^2^.

Figure 5a shows a photograph of the USB 3.0 hub device. An image of the controller chip is shown in the inset. The corresponding THz-ToF results are illustrated in Figure 5b. First, we demonstrate a 3D reconstructed image. A series of layers were identified from top to bottom, indicating the top (*T* = 22 ps) and bottom surfaces (*T* = 40 ps) of the plastic case. More importantly, we can identify the shape and location of the controller chip whose top surface appears at *T* = 50 ps on top of the PCB board surface at *T* = 59 ps. The lateral size of the chip was measured to be 8 × 8 mm^2^, which is expected from a USB 3.0 controller chip. Figure 5c shows a series of ToF images, ToF-3 (top) and ToF-4 (bottom), for the reflected images on the 3rd and 4th reflection layers, respectively. We identified the top surface of the IC chip package (indicated by an arrow) in ToF-3 as a square. Furthermore, we could image the internal structure of the IC package (i.e., the dice surface in the IC chip), as shown in ToF-4. This will provide more solid evidence for the authenticity of IC chips if information on the IC chip structure is known.

We tested another USB hub device containing USB 2.0 controller chips as shown in Figure 5d. This IC chip had a smaller lateral size, as mentioned earlier. As expected, we located the controller chip using THz-ToF imaging, which was placed below the enclosure plastic layers, as shown in the 3D image in Figure 5e. The lateral size was measured to be 4 × 10 mm^2^, which corresponded to a controller chip (USB 2.0). Therefore, the inappropriate use of IC package chips can be easily identified, particularly when they have different sizes. The IC chip was split into two parts because of the thickness difference in the plastic enclosure, resulting in a path length difference between the two regions. In addition, the ToF-3 image shown in Figure 5f allows us to identify the size and location of the top surface of the IC package. Again, the ToF-4 image provided information on the internal structure of the IC chip. Consequently, we conclude that our measurement scheme enables us to identify the inappropriate use of IC chips embedded in plastic packages, and the imaging of the internal structure inside IC-packaged chips will further enhance the possibility of the accurate authentication of electronic devices.

## 4. Conclusions

We demonstrate that THz-ToF imaging is a powerful method for identifying counterfeit electronics embedded in plastic cases by imaging their internal structures in a nondestructive manner. THz-TDS systems with the ASOPS technique enable the rapid imaging of portable electronic devices at 100 Hz/pixel, with a spatial resolution of 1 mm and a depth range larger than 5 mm when a 50 mm scan lens is used. ToF images on portable devices, such as mobile phones, have been obtained, enabling the visualization of internal structures. As an example of authentication, we obtained THz-ToF images on a counterfeit SSD in comparison with an authentic device and observed that there was a striking difference in the internal structures, although their appearances were similar. Authenticating small portable devices, such as earphones, with curved surfaces is also possible using THz-ToF imaging from various viewpoints. Finally, we demonstrate the use of THz-ToF to reveal the inappropriate use of IC chips, such as controller chips, in USB hub devices. We obtained images of the controller chips, which allowed us to determine the physical dimensions of the IC packages. In particular, we could image the internal structure of the packed chips even though the ICs were covered by a thick plastic case. Having a database of THz-TDS images for various portable and wearable devices will be useful for the rapid in-line authentication of products and will offer a unique tool for identifying fake electronics and the inappropriate use of IC chips in various equipment.

## Data Availability

The original contributions presented in this study are included in the article material. Further inquiries can be directed to the corresponding author.
